# Image Feature Fusion of Hyperspectral Imaging and MRI for Automated Subtype Classification and Grading of Adult Diffuse Gliomas According to the 2021 WHO Criteria

**DOI:** 10.3390/diagnostics16030458

**Published:** 2026-02-01

**Authors:** Ya Su, Jiazheng Sun, Rongxin Fu, Xiaoran Li, Jie Bai, Fengqi Li, Hongwei Yang, Ye Cheng, Jie Lu

**Affiliations:** 1 Department of Radiology and Nuclear Medicine, Xuanwu Hospital, Capital Medical University, Beijing 100053, China; 2 Beijing Key Laboratory of Magnetic Resonance Imaging and Brain Informatics, Capital Medical University, Beijing 100053, China; 3 Beijing United Imaging Research Institute of Intelligent Imaging, Beijing 100094, China; 4 School of Precision Instrument and Opto-Electronics Engineering, Tianjin University, Tianjin 300072, China; 5 School of Medical Technology, Beijing Institute of Technology, Beijing 100081, China; 6 Department of Neurosurgery, Xuanwu Hospital, Capital Medical University, Beijing 100053, China

**Keywords:** adult diffuse gliomas, hyperspectral imaging, computer-aided pathology, multimodal fusion framework, magnetic resonance imaging, radiomic features, 2021 WHO types and grades

## Abstract

**Background:** Current histopathology- and molecular-based gold standards for diagnosing adult diffuse gliomas (ADGs) have inherent limitations in reproducibility and interobserver concordance, while being time-intensive and resource-demanding. Although hyperspectral imaging (HSI)-based computer-aided pathology shows potential for automated diagnosis, it often yields suboptimal accuracy due to the lack of complementary spatial and structural tumor information. This study introduces a multimodal fusion framework integrating HSI with routinely acquired preoperative magnetic resonance imaging (MRI) to enable automated, high-precision ADG diagnosis. **Methods:** We developed the Hyperspectral Attention Fusion Network (HAFNet), incorporating residual learning and channel attention to jointly capture HSI patterns and MRI-derived radiomic features. The dataset comprised 1931 HSI cubes (400–1000 nm, 300 spectral bands) from histopathological patches of six major World Health Organization (WHO)-defined glioma subtypes in 30 patients, together with their routinely acquired preoperative MRI sequences. Informative wavelengths were selected using mutual information. Radiomic features were extracted with the PyRadiomics package. Model performance was assessed via stratified 5-fold cross-validation, with accuracy and area under the curve (AUC) as primary endpoints. **Results:** The multimodal HAFNet achieved a macro-averaged AUC of 0.9886 and a classification accuracy of 98.66%, markedly outperforming the HSI-only baseline (AUC 0.9267, accuracy 87.25%; *p* < 0.001), highlighting the complementary value of MRI-derived radiomic features in enhancing discrimination beyond spectral information. **Conclusions:** Integrating HSI biochemical and microstructural insights with MRI radiomics of morphology and context, HAFNet provides a robust, reproducible, and efficient framework for accurately predicting 2021 WHO types and grades of ADGs, demonstrating the significant added value of multimodal integration for precise glioma diagnosis.

## 1. Introduction

Gliomas represent the most prevalent and aggressive group among adult primary brain cancers [1]. In 2021, the World Health Organization (WHO) classification of the central nervous system (CNS) has classified adult diffuse gliomas (ADGs) into three types and graded on a histopathological scale from 2 to 4: (1) astrocytoma, isocitrate dehydrogenase (IDH)-mutant (grade 2, 3, and 4, short for A 2, A 3, A 4), (2) oligodendroglioma with both IDH-mutant and 1p/19q-codeleted (grade 2 and 3, short for O 2, O 3), and (3) glioblastoma with IDH-wildtype (grade 4, short for G 4) [2]. Integrating histology and molecular markers to classify ADGs is essential due to subtype differences in treatment and prognosis [3]. However, diagnostic accuracy is limited by inter-/intra-observer variability and tumor heterogeneity [4,5]. Moreover, the diagnostic process is often time-consuming, labor-intensive, and costly, requiring specialized resources that may not be routinely accessible in clinical settings [6]. There is an urgent need for more reproducible and efficient techniques for the classification and diagnosis of gliomas.

Compared with traditional RGB imaging, hyperspectral imaging (HSI) is an advanced optical modality that offers substantially richer spectral information and higher spectral resolution, serving as a powerful tool for computer-aided pathological diagnosis [7,8]. Owing to its finer spectral details, HSI enhances the performance of pathological diagnosis across various cancers [9,10,11]. Ortega et al. employed a custom 7-layer two-dimensional convolutional neural network (CNN) on 400–1000 nm HSI of hematoxylin and eosin (H&E) brain sections to distinguish glioblastoma from non-tumor tissue, reaching 88% sensitivity and 77% specificity [12]. Chen et al. developed SMLMER-ResNet, a lightweight residual network integrating channel attention and multi-scale spatial–spectral fusion, to classify WHO grades 1–4 in glioma sections, attaining 97.3% accuracy [13]. However, HSI is restricted to two-dimensional histological sections and lacks the ability to capture the macroscopic anatomical and spatial characteristics of tumors. The integration of HSI with complementary imaging modalities may provide a promising strategy to overcome these limitations and enhance the accuracy of glioma classification and grading.

Preoperative medical imaging is an essential component of the diagnostic workflow, providing valuable information for tumor subtype classification, grading, diagnosis, and prognosis [14,15,16]. Numerous studies have shown that incorporating preoperative magnetic resonance imaging (MRI) features into pathological analysis significantly improves diagnostic and prognostic accuracy [17,18,19,20]. Mahootiha et al. developed a multimodal deep-learning model integrating preoperative MRI, clinical data, and molecular profiles to predict post-surgical recurrence in pediatric low-grade gliomas, achieving higher accuracy than conventional methods and enabling earlier, personalized surveillance to reduce both over- and under-treatment [21]. Rathore et al. fused MRI and histopathologic features via Cox and SVC models to predict glioma survival, outperforming single-modality approaches [22]. The integration of MRI with HSI of histopathological specimens for glioma subtype classification and grading holds potential to improve diagnostic accuracy. To date, however, this approach has not been systematically investigated.

In this study, we proposed the Hyperspectral Attention Fusion Network (HAFNet), which integrated HSI features from glioma pathology slides with preoperative MRI radiomic features to accurately predict the 2021 WHO-defined ADG types and grades. We also compared the multimodal fusion strategy with the HSI-only baseline. The proposed fusion model directly addresses the challenge of subjective assessment by providing an automated, reproducible, and efficient approach for computer-aided pathological diagnosis of ADGs, thereby laying a methodological foundation for enhancing diagnostic precision.

## 2. Materials and Methods

### 2.1. Dataset Structure

This retrospective study was approved by our Institutional Review Board (No. 2023-044) with a waiver of informed consent. A total of 53 histologically confirmed glioma patients who underwent preoperative brain MRI and subsequent surgery at Xuanwu Hospital between January 2022 and July 2023 were included. The subjects were excluded as follows: 2 patients with non–ADG diagnosis, 5 patients who had received prior treatment before MRI, 3 patients with inadequate tissue, 3 patients with poor-quality or incomplete MRI sequences, 4 patients with severe systemic or neurological illness, and 6 patients with incomplete clinical data. Eventually, 30 patients were enrolled (mean age, 49 years ± 14 [standard deviation]; 17 male). The inclusion and exclusion criteria are detailed in Supplementary S1.

Tumor specimens resected during surgery were reviewed by experienced pathologists at the Department of Pathology, Xuanwu Hospital, and classified according to the 2021 WHO classification of tumors of the CNS into six types: A 2, 3, and 4; O 2 and 3; and G 4. Detailed clinical information is presented in Supplementary Table S1. The clinical diagnostic workflow is illustrated in Supplementary Figure S1. The clinical pathologists’ diagnoses served as the ground truth in this study.

Hyperspectral data were acquired from pathological tumor sections stained for N-cadherin (NCAD) and counterstained with hematoxylin to visualize nuclei. Details of the immunohistochemical procedure are provided in Supplementary S2. The acquired hyperspectral images were further partitioned into non-overlapping patches.

In total, 1931 hyperspectral patches were obtained, corresponding to the six glioma subtypes: 212 A 2, 302 A 3, 160 A 4, 418 O 2, 343 O 3, and 496 G 4 patches.

### 2.2. Hyperspectral Data Acquisition and Preprocessing

**Figure 1:**
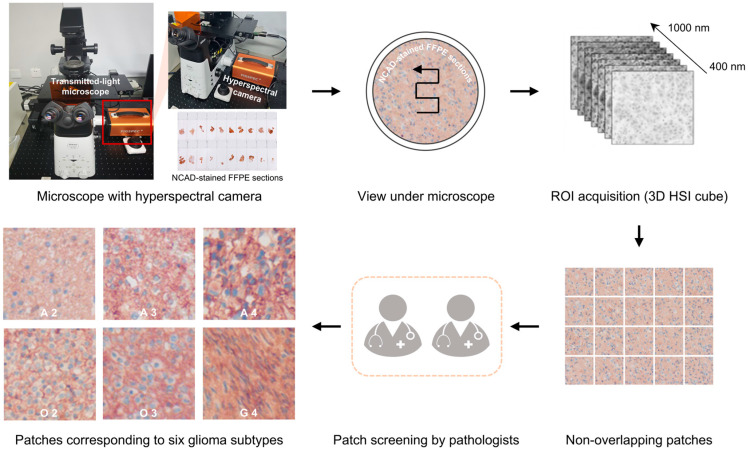
Microscopic hyperspectral data acquisition workflow for six subtypes of glioma pathological sections under a 20× objective lens. NCAD: N-cadherin; FFPE: formalin-fixed paraffin-embedded; ROIs: regions of interest; 3D: three-dimensional; HSI: hyperspectral imaging; A 2/3/4: astrocytoma, IDH-mutant, grade 2/3/4; O 2/3: oligodendroglioma, IDH-mutant and 1p/19q-codeleted, grade 2/3; G 4: glioblastoma, IDH-wildtype, grade 4.

HSIs were acquired using a custom platform integrating a transmitted-light microscope (ECLIPSE Ti, Nikon, Tokyo, Japan) and a hyperspectral camera (FS2X, FigSpec, Hangzhou, China), as shown in Figure 1. The system employs a 20× objective lens and captures spectral data from 400 to 1000 nm with a spectral resolution of 2.5 nm. Each HSI forms a three-dimensional (3D) data cube measuring 534 × 480 pixels spatially, with 300 spectral bands. The pixel size is 5.86 × 5.86 µm. Spectral consistency was ensured through standard white and dark reference calibrations (Supplementary S3).

For each case, at least 10 regions of interest (ROIs) were randomly selected. These ROIs were segmented into non-overlapping patches of 100 × 100 pixels spatially, with 300 spectral bands, for further analysis. Patches with <80% tumor area (i.e., with substantial background) were excluded. Ultimately, patches corresponding to six glioma subtypes were obtained.

### 2.3. MRI Acquisition and Preprocessing

All participants underwent preoperative MRI scans on the United Imaging uPMR 790-integrated PET/MR scanner (Shanghai, China). The MRI protocol encompassed 3D T1-weighted imaging (T1WI), 3D contrast-enhanced T1-weighted imaging (T1CE), diffusion-weighted imaging (DWI), T2-weighted fluid-attenuated inversion recovery (T2 FLAIR), and T2-weighted imaging (T2WI). MRI acquisition parameters are detailed in Supplementary Table S2. An apparent diffusion coefficient (ADC) map was generated from DWI images.

In the MRI preprocessing, N4 bias field correction was performed on all MRI scans to minimize intensity non-uniformities, followed by registration to the T1 image using the open-source software 3D Slicer (version 5.2.1). The co-registration outcomes were checked visually and manually adjusted based on anatomical landmarks when necessary. Subsequently, glioma segmentation for radiomics analysis was performed by two board-certified neuroradiologists who manually delineated the whole-tumor ROI on the T2 FLAIR sequences using ITK-SNAP software (version 4.2.0). Any inter-observer discrepancies were resolved through consensus review.

### 2.4. MRI Analysis

Radiomic features were obtained from both the original preprocessed MR images and images processed with Laplacian of Gaussian (LoG) filters at σ values of 1, 2, and 3, as well as from first-level wavelet decompositions using the Haar wavelet. Using the open-source PyRadiomics package (version 3.0.1), a total of 5650 radiomic features were extracted for each patient, encompassing shape, first-order, texture, LoG-filtered, and wavelet-transformed features across five imaging sequences (T1WI, T1CE, T2 FLAIR, T2WI, and ADC). Shape features, which capture the tumor’s geometric and morphological properties, were derived exclusively from the original preprocessed MR images. First-order features, characterizing the distribution of voxel intensities within the tumor, and texture features—including gray-level cooccurrence matrix (GLCM), gray-level dependence matrix (GLDM), gray-level run length matrix (GLRLM), neighboring gray tone difference matrix (NGTDM), and gray-level size zone matrix (GLSZM)—were extracted from both the original and filtered images.

### 2.5. Mutual Information-Based Wavelength Selection

**Figure 2:**
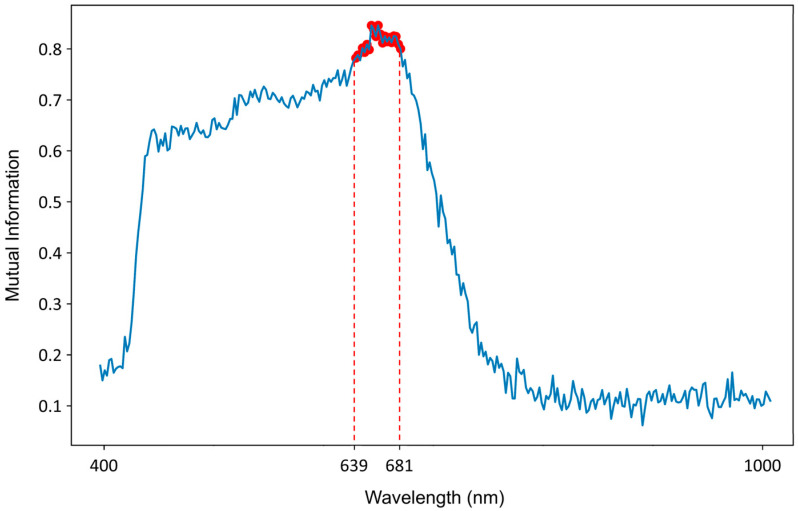
Mutual information-based selection of the top 20 informative wavelengths for glioma classification. The selected top 20 wavelengths (639–681 nm) are highlighted with a red outline.

To identify the most discriminative wavelengths for spectral data classification, we employed mutual information (MI) as a feature selection criterion. MI quantifies the statistical dependence between two random variables by measuring the reduction in uncertainty of one variable given knowledge of the other, formally defined as:

(1) $$I\left(X;Y\right)=H\left(X\right)-H\left(X|Y\right)=\sum_{x,y}p\left(x,y\right)\log\frac{p\left(x,y\right)}{p\left(x\right)p\left(y\right)}$$

where $H\left(X\right)$ is the entropy of X, $H\left(X|Y\right)$ is the conditional entropy, and $p\left(x,y\right)$, $p\left(x\right)$, $p\left(y\right)$ are the joint and marginal probability distributions, respectively. In this study, MI scores were computed between the mean spatial intensity at each wavelength and the class labels using the scikit-learn implementation (mutual_info_classif). The top 20 wavelengths (639–681 nm) were selected (Figure 2) to maximize class separability and minimize redundancy, enhancing the robustness of the downstream CNN.

### 2.6. Hyperspectral Attention Fusion Network

#### 2.6.1. Overview

**Figure 3:**
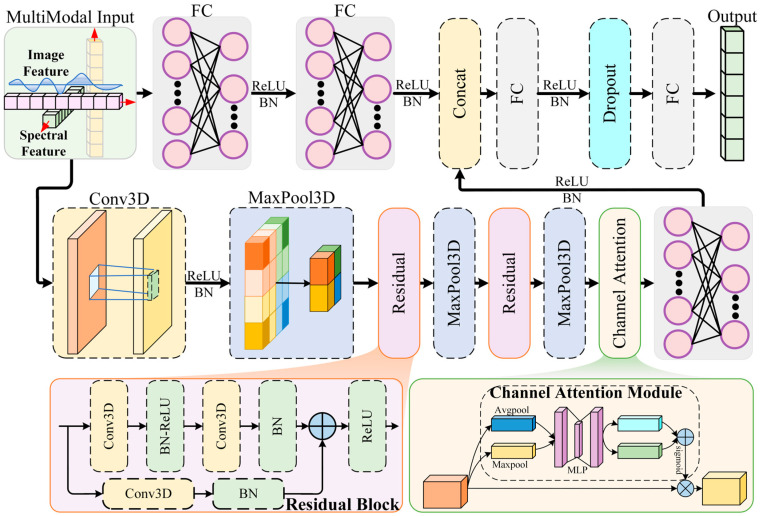
Schematic overview of the proposed HAFNet architecture that integrates residual learning, channel attention, and multimodal fusion. FC: fully connected; ReLU: rectified linear unit; BN: batch normalization; Conv3D: three-dimensional convolution; MaxPool3D: three-dimensional max pooling; Avgpool: average pooling; MLP: multi-layer perceptron.

We propose HAFNet, a novel 3D CNN architecture that synergistically integrates residual learning, channel attention, and multimodal fusion for robust glioma subtyping into six WHO 2021 classes (Figure 3). It employs attention-enhanced residual blocks to emphasize discriminative spectral channels and a dedicated fusion module to combine HSI and MRI features, markedly improving classification accuracy. The network processes HSI inputs of shape (N, 1, C, H, W)—where N is the batch size, C is the number of selected wavelengths, and H = W = 100 represent spatial dimensions—alongside MRI feature vectors of 5650 dimensions, outputting class probabilities via softmax.

#### 2.6.2. Convolutional Backbone with Residual Connections

The backbone begins with an initial 3D convolutional layer to extract low-level spatio-spectral features, using a kernel size of (5, 3, 3), stride of (2, 1, 1), and padding of (2, 1, 1), followed by batch normalization (BN), rectified linear unit (ReLU) activation, and max pooling with a kernel of (2, 2, 2). This step reduces the input dimensions while capturing essential patterns, resulting in an output shape of (N, 32, C/2, H/2, W/2).

To enable deeper architectures without issues like gradient vanishing, we incorporate two residual blocks inspired by ResNet principles. Each block consists of two 3D convolutional layers (kernel size = 3, padding = 1) with BN and ReLU activation, plus a shortcut connection that adds the input directly to the output for efficient information flow. If channel dimensions or strides differ, a 1 × 1 convolution adjusts the shortcut path. The first block expands channels from 32 to 64, followed by max pooling (kernel = 2, 2, 2), yielding (N, 64, C/4, H/4, W/4). The second block expands to 128 channels with pooling (kernel = 1, 2, 2), resulting in (N, 128, C/4, H/8, W/8). These residuals allow HAFNet to learn hierarchical features from high-dimensional HSI data, which is particularly effective for identifying glioma-specific spectral patterns such as absorption peaks.

#### 2.6.3. Channel Attention Mechanism

After the residual blocks, a channel attention module adaptively recalibrates the feature maps to emphasize informative spectral channels. This is achieved by computing global average and max pooling on the input features, passing them through shared fully connected (FC) layers with a reduction ratio of 16 and ReLU activation, then combining the results and applying a sigmoid function to generate channel-wise weights. These weights are multiplied element-wise with the original features, enhancing focus on tumor-discriminative channels (those with high mutual information scores) while suppressing noise from irrelevant wavelengths. Applied after the second residual block, this module improves the model’s ability to discern subtle spectral signatures.

#### 2.6.4. Multimodal Fusion Branch

HAFNet’s fusion mechanism integrates HSI features with MRI-derived radiomic features. HSI features are first flattened (~8192 dimensions after the convolutional backbone) and projected to 512 dimensions via an FC layer with BN and ReLU activation. Simultaneously, MRI features are processed through two FC layers with BN and ReLU, reducing their dimensionality to 256. The two feature sets are then concatenated (768 dimensions) and fed to a fusion FC layer that outputs 512 dimensions, followed by BN, ReLU, and dropout with a rate of 0.3 to prevent overfitting. This design ensures complementary integration: HSI provides biochemical insights while MRI adds structural context.

#### 2.6.5. Training and Optimization

HAFNet is trained end-to-end using weighted cross-entropy loss to handle class imbalance, with weights calculated based on balanced class distributions. Optimization employs the Adam algorithm with a learning rate of 1 × 10^−4^ and weight decay of 10^−4^, along with ReduceLROnPlateau scheduling (factor = 0.5, patience = 5) and early stopping (patience = 5 epochs). The dataset was randomly partitioned into training and testing sets at an 80:20 ratio. To ensure robustness and generalizability, we performed 5-fold cross-validation on the training set, where each fold maintained the same proportion of training and validation data. Data augmentation includes random flips, 90° rotations applied per wavelength channel, and brightness scaling between 0.8 and 1.2. Wavelength selection retains the top 20 channels based on mutual information, reducing input dimensionality by 93.3% while preserving discriminative power.

## 3. Results

### 3.1. Area Under the Receiver Operating Characteristic Curve (ROC–AUC) Comparison Between HSI-Only and Multimodal Configurations

To assess the impact of multimodal integration, we conducted experiments using both single-modality (HSI-only) and multimodal (HSI fused with MRI features) configurations. Empirical evaluations on our glioma dataset, comprising HSI scans and corresponding MRI-derived radiomic features from 30 cases, demonstrate HAFNet’s superior performance across multiple metrics.

**Figure 4:**
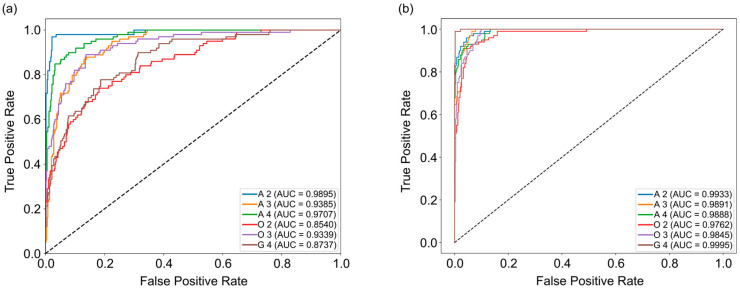
Comparison of ROC-AUC between single-modality (HSI-only, (**a**)) and multimodal HAFNet (fusion of HSI and MRI features, (**b**)) settings for glioma classification. The fusion model demonstrated consistently higher AUC across all six WHO-defined glioma classes. A 2/3/4: astrocytoma, IDH-mutant, grade 2/3/4; O 2/3: oligodendroglioma, IDH-mutant and 1p/19q-codeleted, grade 2/3; G 4: glioblastoma, IDH-wildtype, grade 4.

The macro-averaged AUC in the multimodal setting reached 0.9886, representing a substantial improvement over the HSI-only AUC of 0.9267 (Figure 4). This indicates enhanced discriminative capability across all six glioma subtypes. The results were derived from stratified 5-fold cross-validation to ensure robustness against data variability, with statistical significance validated using paired *t*-tests (*p* < 0.001).

On a per-class basis, improvements are evident across all glioma subtypes: A 2 (0.9933 vs. 0.9895), A 3 (0.9891 vs. 0.9385), A 4 (0.9888 vs. 0.9707), O 2 (0.9762 vs. 0.8540), O 3 (0.9845 vs. 0.9339), and G 4 (0.9995 vs. 0.8737), suggesting that MRI-derived features effectively complement HSI, thereby improving subtype discrimination in heterogeneous tumor presentations.

### 3.2. Classification Accuracy Comparison Between HSI-Only and Multimodal Configurations

**Figure 5:**
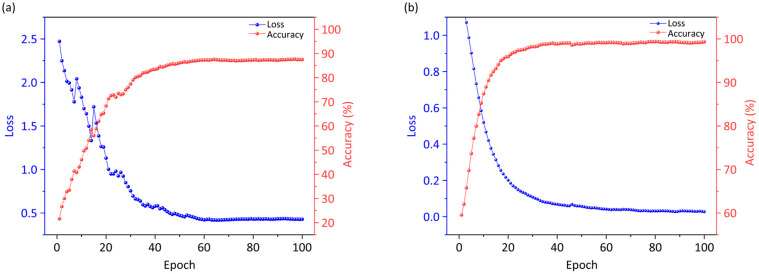
Loss and accuracy curves over 100 epochs for single-modality (**a**) configurations and multimodal (**b**) configurations.

The training dynamics are visualized in Figure 5 with test loss and accuracy over 100 epochs. In the single-modality case (Figure 5a), the test loss decreases rapidly from an initial value near 2.5, stabilizing around 0.4 after approximately 60 epochs, indicating effective but gradual convergence. The test accuracy increases steadily and eventually plateaus at 87.25%. In contrast, the multimodal configuration (Figure 5b) demonstrates superior dynamics, with the test loss dropping sharply to below 0.05 within 50 epochs and exhibiting minimal fluctuations thereafter. The test accuracy ascends more rapidly, achieving a final value of 98.66%—an 11.41% improvement over the single-modality baseline. These notable improvements demonstrate the efficacy of integrating hyperspectral information with MRI-derived features, thereby reinforcing feature representation and enhancing class separability.

### 3.3. Confusion Matrix Analysis: Performance Comparison Between HSI-Only and Multimodal Configurations

**Figure 6:**
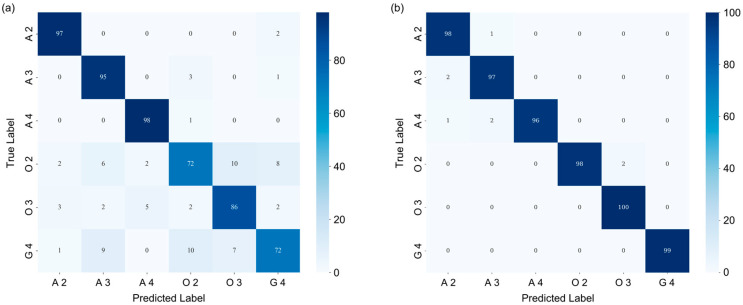
Confusion matrices for glioma subtype classification using HSI-only (**a**) and multimodal (**b**) approaches. Color bars represent patch counts, where intensity increases with higher values. A 2/3/4: astrocytoma, IDH-mutant, grade 2/3/4; O 2/3: oligodendroglioma, IDH-mutant and 1p/19q-codeleted, grade 2/3; G 4: glioblastoma, IDH-wildtype, grade 4.

Further insights are provided by the confusion matrix analysis, as shown in Figure 6. The matrices are 6 × 6 grids with rows representing true labels and columns representing predicted labels for glioma subtypes: A 2, A 3, A 4, O 2, O 3, and G 4. Following class-balance processing, the test set contained 99, 99, 99, 100, 100, and 99 patches for these subtypes, respectively. Values in the confusion matrix show the distribution of patches across the predicted categories, with a color gradient from light blue (low) to dark blue (high).

For the HSI-only matrix (Figure 6a), diagonal elements indicate reasonable but variable correct classifications, ranging from 72 (O 2 and G 4) to 98 (A 4). Off-diagonal errors are pronounced, especially between similar grades (e.g., O 2 misclassified as O 3 (10), and G 4 as A 3 (9)). This reveals HSI’s limitations in resolving subtle spectral overlaps, resulting in a macro F1-score of ~0.87.

In contrast, the multimodal matrix (Figure 6b) exhibits strong diagonal dominance, with the number of correct predictions ranging from 96 (A 4) to 100 (O 3), corresponding to accuracies all above 96%. Off-diagonal misclassifications are substantially reduced (e.g., O 2 as O 3 (2), and G 4 as A 3 (0)). This yields a macro F1-score of ~0.99, with errors reduced by 9× compared to HSI-only, highlighting fusion’s role in enhancing specificity for challenging subtypes like O 2 and O 3.

## 4. Discussion

In this study, we proposed a multimodal deep learning model (HAFNet) integrating HSI features with preoperative MRI radiomic features to achieve accurate subtype classification and grading of ADGs strictly according to the 2021 WHO rule. Compared with the HSI-only model, the fusion model demonstrated significant improvements in diagnostic accuracy and robustness, validating the great potential of multi-modal imaging integration in computer-aided glioma diagnosis.

Both HSI and MRI have been extensively validated as valuable modalities in the precise diagnosis of gliomas. Ortega et al. used HSI and supervised classifiers to distinguish glioma from normal brain tissue on slides, reaching ~75% across-patient accuracy, demonstrating HSI’s potential as an adjunct for pathological diagnosis [23]. Chen et al. employed HSI and the SMLMER-ResNet CNN to grade glioma malignancy on 296 WHO-classified slides, reaching 99.7% overall accuracy [13]. Xin et al. used stimulated-Raman HSI to decode lipid-/protein-associated metabolic fingerprints of gliomas across WHO grades 1–4, achieving 92.6% accuracy via a CNN model [24]. Zhu et al. leveraged the rich spectral–spatial information of label-free HSI to train a Pix2Pix-Unet network, enabling direct conversion of unstained glioma tissue into high-fidelity virtual H&E stains without chemical processing [25]. These studies highlight the considerable potential of HSI for accurate glioma detection and grading; however, as a standalone modality, it remains insufficient for delivering the integrated molecular–morphological characterization required by the latest WHO classification, resulting in incomplete classification and suboptimal accuracy.

Due to the exceptional capacity for delineating anatomical structures and characterizing tissue textures, MRI has been widely applied in glioma diagnosis. Zhou et al. developed a non-invasive approach for preoperative glioma grading using contrast-enhanced T1WI [26]. Fan et al. highlighted the role of AI-driven MRI radiomics and radiogenomics in non-invasively predicting glioma grade, molecular subtypes, and patient prognosis [27]. Lin et al. used radiomic features from synthetic-MRI quantitative maps and machine-learning classifiers to predict WHO grades and molecular subtypes of diffuse gliomas, achieving AUC values of 0.90–0.94 [28]. Li et al. leveraged MRI radiomics and machine learning to estimate glioma survival and macrophage infiltration, achieving a concordance index of 0.81 [29]. In our controlled experiments, an MRI-only baseline model achieved a macro-AUC of 0.8980 and an accuracy of 83.89% (see Supplementary S4, Figure S2), which, while competent, was notably lower than our multimodal fusion model. Given that histopathological examination remains the gold standard for glioma diagnosis, MRI-based findings often require pathological validation to enhance their clinical reliability and interpretability, underscoring the importance of exploring complementary imaging modalities for synergistic integration. There remains a lack of comprehensive analysis regarding the synergistic potential of multimodal fusion between HSI and MRI in existing research.

In this work, the combination of HSI’s rich spectral information with the structural and functional imaging advantages of MRI enables the multimodal approach to capture tumor heterogeneity from multiple dimensions, achieving superior performance compared with previous studies (accuracy 98.66%, macro-averaged AUC 0.9886). The robustness of these results is evidenced by low cross-validation variance (AUC SD: 0.0041; Accuracy SD: 0.0033) and stable learning curves, indicating minimal overfitting. HSI reflects cellular and tissue biochemical composition and microstructural alterations through meticulous spectral dimensions, thereby complementing the limitations of traditional histopathology and morphological analysis. This capability is visually affirmed by model attention maps (Supplementary S5, Figure S3), which demonstrate HAFNet’s focused response to histopathologically relevant features—including nuclear morphology and molecular distribution patterns—within the HSI spectra. MRI offers macroscopic anatomical and spatial distribution characteristics of tumors, encompassing tumor morphology, texture heterogeneity, and the microenvironmental context. The complementarity of these modalities allows the fusion model to comprehensively identify distinguishing characteristics of glioma subtypes, thereby enhancing classification accuracy in accordance with the 2021 WHO CNS glioma framework, which emphasizes integrated imaging diagnostics encompassing both molecular information and morphological assessment.

In computer-aided pathology analysis of H&E-stained slides, cutting-edge artificial intelligence models tend to focus on nuclei for their high contrast, clear boundaries, and strong diagnostic relevance, whereas the cytoplasm—pink from eosin bound to acidophilic proteins and organelles—represents an averaged mixture of constituents and thus offers limited specific diagnostic value [8,30,31]. In this study, NCAD immunohistochemical slides, subjected to hematoxylin counterstaining, were employed, preserving essential nuclear morphological and textural features while specifically labeling the single protein NCAD. NCAD is a calcium-dependent cell adhesion molecule that exhibits differential expression among glioma subtypes and is associated with tumor grade and invasive potential [32,33,34,35]. HSI offers the unique capability to non-destructively and quantitatively analyze the biochemical composition and content of tissues, enabling precise measurement of NCAD positivity within slides. When combined with nuclear features, this facilitates accurate differentiation of pathological slides across glioma subtypes. However, whether hyperspectral analysis of NCAD-stained slides provides a significant advantage over H&E-stained slides—and the magnitude of such potential benefits—remains to be experimentally validated. Moreover, a trade-off may exist between the convenience of staining procedures and diagnostic precision. This work presents a multimodal, automated computer-aided pathology analysis framework integrating HSI with preoperative MRI. The applicability and diagnostic accuracy of this approach for more widely used H&E-stained slides, unlabeled fresh tumor tissues, and even intraoperative in vivo tumor specimens will constitute important directions for future research.

Nonetheless, certain limitations remain. The primary one is the modest sample size (n = 30) for a six-class task, which may impact model robustness and generalizability. Future work could address these issues by incorporating self-supervised pretraining on larger, multi-center datasets. Additionally, while the NCAD-based analysis demonstrates feasibility, the framework’s applicability to other immunohistochemical markers, conventional H&E slides, and intraoperative fresh tissue remains to be established. Finally, improving model interpretability will be important for enhancing clinicians’ understanding of, and trust in, the algorithmic decision-making process.

## 5. Conclusions

In this study, we developed the HAFNet, which integrates HSI features from NCAD-stained glioma pathology slides with preoperative MRI radiomic features to enable accurate classification of ADGs in accordance with the 2021 WHO CNS criteria. This multimodal fusion strategy harnesses the rich spectral–biochemical sensitivity of HSI and the macroscopic anatomical and structural characterization capabilities of MRI, capturing complementary information across microscopic and macroscopic scales. The proposed approach achieved a substantial improvement over the HSI-only baseline, attaining a macro-averaged AUC of 0.9886 and an accuracy of 98.66%. To the best of our knowledge, this represents a novel attempt to integrate HSI and MRI for glioma subtype classification, delivering an automated and reproducible approach with high diagnostic accuracy that could contribute to objective and standardized diagnostic workflows for glioma characterization.

## Data Availability

The datasets used and analyzed during the current study are available from the corresponding author on reasonable request.

## Supplementary Materials

The following supporting information can be downloaded at: https://www.mdpi.com/article/10.3390/diagnostics16030458/s1, Figure S1: Schematic of the integrated classification pipeline for adult diffuse gliomas according to the 2021 WHO criteria; Figure S2: Performance of the MRI-only baseline model; Figure S3: Visualization of model attention on an HSI patch; Table S1: Patients characteristics; Table S2: Acquisition parameters for MRI.
