# Supplementary material for: Image Feature Fusion of Hyperspectral Imaging and MRI for Automated Subtype Classification and Grading of Adult Diffuse Gliomas According to the 2021 WHO Criteria
Source: Diagnostics (Basel). 2026 Feb 1;16(3):458. doi: 10.3390/diagnostics16030458 (PMC12897174; doi:10.3390/diagnostics16030458)
Supplement: Supplementary file 1 [file diagnostics-16-00458-s001.zip › diagnostics-4050668-supplementary.pdf]

S1: Inclusion and exclusion criteria

Inclusion criteria: 1) Patients diagnosed with adult diffuse gliomas confirmed by histopathology. 2) Patients who have undergone preoperative brain magnetic resonance imaging (MRI) containing routinely sequences with sufficient quality (T1WI, T1CE, T2WI, T2 FLAIR, DWI). 3) Availability of sufficient tumor tissue to allow complete histopathological diagnosis and immunohistochemical (IHC) staining. 4) Complete clinical and pathological diagnostic information, including tumor subtype and grade as defined by the 2021 World Health Organization (WHO) classification of tumors of the central nervous system (CNS). 5) Age between 18 and 80 years.

Exclusion criteria: 1) Inadequate tumor tissue or failed staining procedures that precluded hyperspectral data acquisition. 2) Severe motion artifacts, low resolution, or incomplete sequences in MRI scans that precluded reliable radiomic analysis. 3) Histopathological diagnosis other than adult diffuse gliomas (e.g., pilocytic astrocytoma, ependymoma, or other CNS tumors). 4) History of neurosurgical resection, radiotherapy, or chemotherapy before preoperative MRI or tissue sampling. 5) Severe systemic illness or neurological disease that could confound MRI findings or pathological assessment. 6) Incomplete histopathological, clinical, or imaging data. 7) A time interval exceeding 2 weeks between preoperative MRI and surgical tumor resection.

S2: IHC staining of N-cadherin (NCAD) in glioma tissue

The tissue sections are derived from surgically resected specimens of the preoperative

MRI-defined tumor volumes. For multifocal cases, intraoperative numbering provided a verifiable chain of custody from each MRI focus to its corresponding pathological slide, establishing lesion-level correspondence for multimodal analysis. Glioma tissue blocks fixed in formalin and embedded in paraffin were sliced into 4 μm thick sections using a microtome. Immunohistochemical analysis for NCAD was conducted employing a horseradish peroxidase (HRP)-linked detection system, with 3,3′-diaminobenzidine (DAB) serving as the chromogenic substrate. The protocol involved initial deparaffinization and rehydration steps, followed by antigen retrieval via heating in citrate buffer (pH 6.0) at temperatures between 95 and 100°C for 20 minutes. After cooling, endogenous peroxidase activity was quenched by treatment with 3% hydrogen peroxide. Sections were incubated overnight at 4°C with a primary antibody targeting NCAD, and then exposed to an HRP-conjugated secondary antibody for 30 minutes at room temperature. The application of DAB resulted in a brown coloration marking positive NCAD expression areas. Subsequently, hematoxylin counterstaining was performed to stain nuclei blue-purple, enhancing tissue morphology visualization. Two independent pathologists reviewed all stained slides to ensure the staining quality. For slides containing both cancerous and normal tissues, pathologists manually annotated the cancerous regions to delineate specific areas for subsequent hyperspectral data acquisition.

S3: Hyperspectral data calibration and preprocessing

Prior to hyperspectral imaging (HSI), white balance calibration of the hyperspectral camera was performed using a blank area on the slide without tissue or dust, under the

same focus settings and illumination as the slide scanning. Then, a HSI of this region was captured to serve as the white reference. The dark balance for the hyperspectral camera was obtained by blocking the objective lens, while the hyperspectral camera automatically acquired the dark reference image during tissue image acquisition.

To minimize system noise, all hyperspectral images of the slides were calibrated with the white and dark reference images, as follows:

$$I(\lambda) = \frac{I_{raw}(\lambda) - I_{dark}(\lambda)}{I_{white}(\lambda) - I_{dark}(\lambda)} \qquad \text{(S1)}$$

where $I(\lambda)$ denotes the normalized transmittance data at wavelength λ, $I_{raw}(\lambda)$ represents the intensity value of the raw hyperspectral image, and $I_{dark}(\lambda)$ and $I_{white}(\lambda)$ refer to the intensity values of the dark and white reference images, respectively. This procedure ensures the consistency of the data and the reproducibility of the algorithmic results.

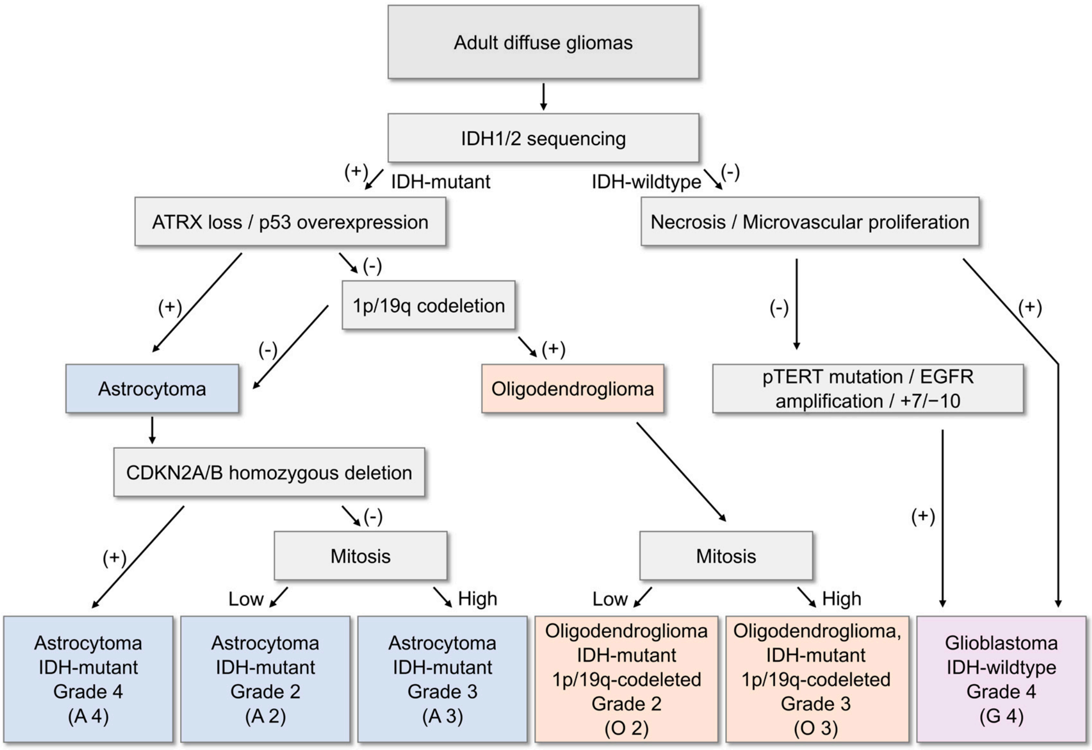

Fig. S1. Schematic of the integrated classification pipeline for adult diffuse gliomas according to the 2021 WHO criteria. IDH: isocitrate dehydrogenase; ATRX: alpha-thalassemia/mental retardation syndrome X-linked; CDKN2A/B: cyclin dependent kinase inhibitor 2A and 2B; pTERT: telomerase reverse transcriptase promoter; EGFR: epidermal growth factor receptor.

Table S1 Patients characteristics

2021 WHO types	Gender		Age		Weight (kg)	
	Male	Female	Mean ± SD	Range	Mean ± SD	Range
A 2	1	2	46 ± 13	31–54	82 ± 12	75–96
A 3	4	1	53 ± 16	31–71	73 ± 14	62–96
A 4	2	1	43 ± 16	31–61	79 ± 15	66–96
O 2	3	5	41 ± 14	25–63	65 ± 15	53–98
O 3	2	2	40 ± 9	34–54	66 ± 16	46–86
G 4	5	2	63 ± 4	56–69	71 ± 6	60–76

WHO: world health organization; SD: standard deviation; A 2/3/4: astrocytoma, IDH-mutant, grade 2/3/4; O 2/3: oligodendroglioma, IDH-mutant and 1p/19q-codeleted, grade 2/3; G 4: glioblastoma, IDH-wildtype, grade 4.

Table S2 Acquisition parameters for MRI

MR sequences	TE (ms)	TR (ms)	Thickness (mm)	FOV (mm)	Matrix
3D T1WI	3	7.2	1.0	256×256	256×100
3D T1CE	3	7.2	1.0	256×256	256×100
DWI b=1000	100.6	2473	5.0	256×255	192×100
T2 FLAIR	100.4	8000	5.0	256×200	256×100
T2WI	104.04	5181	5.0	256×200	256×100

MRI: magnetic resonance imaging; TE: echo time; TR: repetition time; FOV: field of view; 3D: three-dimensional; T1WI: T1-weighted imaging; T1CE: contrast-enhanced T1-weighted imaging; DWI: diffusion-weighted imaging; T2 FLAIR: T2-weighted fluid-attenuated inversion recovery; T2WI: T2-weighted imaging.

S4: Performance of the MRI-only baseline model

To establish a complete control baseline, we evaluated the performance of an MRI-only configuration using the same network architecture and training protocol, with only MRI-derived radiomic features as input. The macro-averaged area under the receiver operating characteristic curve (AUC) for the MRI-only model was 0.8980. The per-class AUCs were as follows: astrocytoma, IDH-mutant, grade 2 (A 2, 0.9732), grade 3 (A 3, 0.9287), grade 4 (A 4, 0.9389), oligodendroglioma, IDH-mutant and 1p/19q-codeleted, grade 2 (O 2, 0.8137), grade 3 (O 3, 0.8807), and glioblastoma, IDH-wildtype, grade 4 (G 4, 0.8526) (Fig. S2a). This model achieved an overall classification accuracy of 83.89%.

The confusion matrix analysis in Fig. S2b reveals further insights. It demonstrates a moderate diagonal concentration, where correct predictions range from 69 (O 2 and G 4) to 94 (A 2 and A 4). However, pronounced off-diagonal errors are observed, particularly the misclassification of G 4 as O 2 (12), O 2 as O 3 (10) and G 4 as A 3 (9), highlighting the model's specific challenges in distinguishing between certain grades and phenotypes.

These results demonstrate that the MRI-only model is consistently inferior to the HSI-only model across all metrics (HSI-only AUC: 0.9267, Accuracy: 87.25%), and is vastly outperformed by the multimodal HAFNet (AUC: 0.9886, Accuracy: 98.66%). This controlled experiment definitively confirms that the superior performance of our proposed model stems from the effective fusion of complementary information from

both HSI and MRI modalities, rather than from the dominance of a single modality.

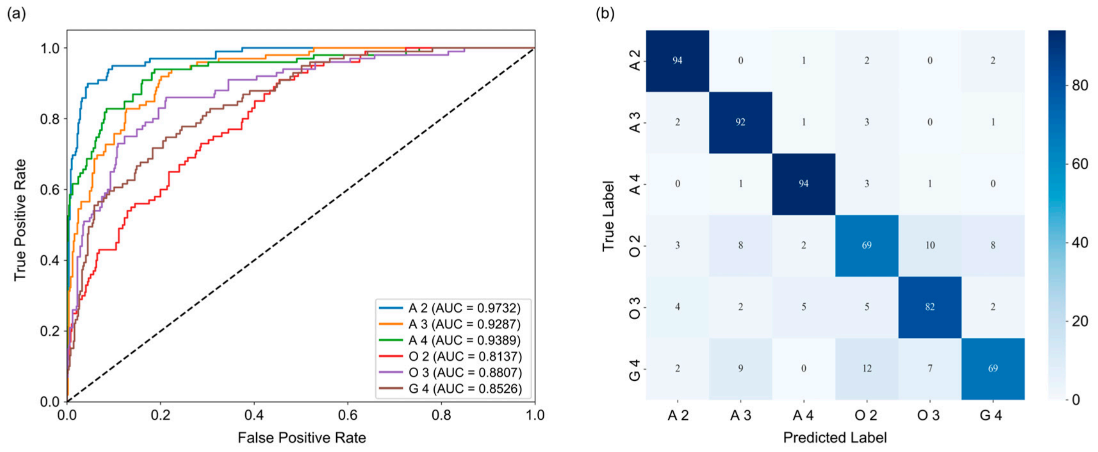

Fig. S2. Performance of the MRI-only baseline model. (a) Receiver operating characteristic curves (macro-averaged AUC = 0.8980). (b) Confusion matrix. Colorbars represent patch counts, where intensity increases with higher values. A 2/3/4: astrocytoma, IDH-mutant, grade 2/3/4; O 2/3: oligodendroglioma, IDH-mutant and 1p/19q-codeleted, grade 2/3; G 4: glioblastoma, IDH-wildtype, grade 4.

S5: Visualization of model attention in HSI patches

To elucidate the decision-making process of HAFNet, we provide a visualization of the model's attention on representative HSI patches (Fig. S3). The heatmap is produced by extracting the feature maps from the final convolutional layer of HAFNet. We compute the mean across the channel dimension to aggregate regions of high activation, then upsample and normalize this aggregation to the original image size. The resulting heatmap intuitively highlights the pathological regions that the model prioritizes during classification. As illustrated, areas with high activation (shown in red and yellow) are predominantly concentrated in regions with dense cell nuclei and areas corresponding

to high NCAD protein expression. Moreover, the morphology of these high-response regions shows a strong correlation with the spatial distribution and shape of the cell nuclei. This visualization effectively validates the interpretability of our model, demonstrating that HAFNet learns to focus on biologically relevant morphological structures and molecular distribution features within the HSI data, rather than on irrelevant background noise.

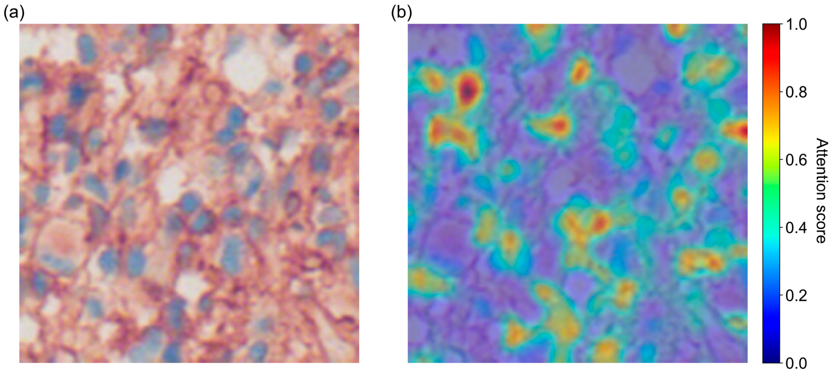

Fig. S3. Visualization of model attention on an HSI patch. (a) A representative raw HSI microscopy patch from a glioma tissue section. (b) The corresponding attention heatmap generated by HAFNet, overlaid on the original patch. The color gradient from blue to red indicates increasing model activation, highlighting regions (e.g., cell nuclei clusters) that most contributed to the classification decision.
